# ‘Challenges and opportunities for imaging on a global scale’ special collection—introductory editorial

**DOI:** 10.1093/bjro/tzaf023

**Published:** 2025-09-25

**Authors:** Erasmo de la Peña, Michael Jackson, Sonal Krishan, Ntobeko Ntusi, Sarah Sheard

**Affiliations:** TecSalud, Tecnologico de Monterrey University Hospitals, Zambrano Hellion Hospital, Monterrey, Nuevo Leon 64753, Mexico; Imaging Department, Royal Hospital for Children and Young People, Edinburgh, EH16 4TJ, United Kingdom; Department of Radiology, Medanta Hospital, Gurgaon, 122018, India; South African Medical Research Council, 7500, South Africa; Imaging Department, Imperial College Healthcare NHS Trust, London, W2 1NY, United Kingdom

Globally, 4.5 billion people lack access to diagnostic imaging.^1^ It is not uncommon that in low- and middle-income countries and, in fact, pockets in high-income countries, many rural clinics are equipped with machines but lack trained technologists as well as radiologists, cardiologists or other specialists to operate them. This is not merely a logistical shortcoming–it is a moral failure. The potential of medical imaging to alleviate suffering—through early detection, accurate diagnosis, and timely intervention–is useless if only confined to urban hubs or high-income countries. Global imaging inequality is further exacerbated by a tendency to over image in well-resourced centers, often for conditions that would be better addressed by preventative measures than costly imaging. Most imaging specialists working in a high-income setting would also acknowledge a significant proportion of their imaging workload is of doubtful clinical benefit to the patient, but performed for fear of litigation, questionable protocols, or as a means to expedite discharge from the emergency department.^2–4^ Rebalancing radiology resources may confront some of us with the need to consider doing less imaging, not more.^5–8^

Encouragingly, some solutions are already within reach:

- Democratising expertise through task-sharing, teleradiology, and decentralised training models.
- AI as an equaliser, designed not to replace radiologists in resource-rich centres, but to support clinicians where none exist.
- Low-cost, high-impact tools, such as portable ultrasound, low field MRI and simplified acquisition protocols and reporting systems, tailored to frontline settings.
- Distantly accessed MR and CT systems to teach and guide acquisitions in real-time maintaining quality assurance.

Yet equity alone is not enough. As we push forward, we must address the environmental impact of medical imaging. Healthcare is responsible for 4.4% of global greenhouse gas emissions, with imaging a significant contributor. A single MRI can consume as much electricity as an average household uses in three months. In drought-prone regions, the water and energy costs of imaging become even more critical. Justification of imaging can no longer be confined to the narrow risk: benefit calculation of the patient being examined.^9^^,^^10^

We must advocate for:

- Green imaging protocols
- Renewable energy integration in radiology infrastructure
- Sustainable procurement and decommissioning practices

Sustainable radiology is not optional—it is our duty to future generations.

We are now at a pivotal moment. The convergence of AI, accessibility, and sustainability demands a new breed of imaging specialist. This profession blends technical mastery with ethical stewardship, leveraging tools not for prestige but for planetary and patient well-being. An imaging specialist’s choices–whether to embrace ethical AI, support rural colleagues, or reduce environmental impact–can carry global implications.

As co-Guest Editors of this special collection in *BJR*|*Open*, we invite our colleagues around the world to reimagine the purpose of medical imaging. Let us move from the ‘I’ of individual achievement to the ‘we’ of shared responsibility. We hope to see high-quality original research and review manuscripts submitted in the following areas:

- Running imaging and radiation oncology services in low-middle income countries
- Trauma imaging in war zones, including the practicalities of imaging itself plus teamwork, managing mental wellbeing and more.
- Imaging prevalent diseases in low-middle income countries
- Sustainability
- AI for the developing world

We hope this carefully curated collection will grow and develop over time, generate interest in the community, and lead to a paradigm shift. We look forward to seeing your submissions and hope you enjoy reading the articles.
